# Supplementary material for: LNP-RNA-engineered adipose stem cells for accelerated diabetic wound healing
Source: Nat Commun. 2024 Jan 25;15:739. doi: 10.1038/s41467-024-45094-5 (PMC10811230; doi:10.1038/s41467-024-45094-5)
Supplement: Supplementary file 1 — Supplementary information [file 41467_2024_45094_MOESM1_ESM.pdf]

LNP-RNA-engineered adipose stem cells for accelerated diabetic wound healing

Yonger Xue[1,12], Yuebao Zhang[1,12,] Yichen Zhong[1,2,12], Shi Du[1], Xucheng Hou[2], Wenqing Li[1], Haoyuan Li[2], Siyu Wang[2], Chang Wang[2], Jingyue Yan[1], Diana D. Kang[1], Binbin Deng[3], David W. McComb[3,4], Darrell J. Irvine[5,6,7,8,9], Ron Weiss[5,10,11], Yizhou Dong[1,2,13*].

1. Division of Pharmaceutics & Pharmacology, College of Pharmacy, The Ohio State University, Columbus, OH, USA.
2. Icahn Genomics Institute, Precision Immunology Institute, Department of Immunology and Immunotherapy, Department of Oncological Sciences, Tisch Cancer Institute, Friedman Brain Institute, Biomedical Engineering and Imaging Institute, Icahn School of Medicine at Mount Sinai, New York, NY, USA.
3. Center for Electron Microscopy and Analysis, The Ohio State University, Columbus, OH, USA.
4. Department of Materials Science and Engineering, The Ohio State University, Columbus, OH, USA.
5. Department of Biological Engineering, Massachusetts Institute of Technology, Cambridge, MA, USA.
6. Koch Institute for Integrative Cancer Research, Massachusetts Institute of Technology, Cambridge, MA, USA.
7. Department of Materials Science and Engineering, Massachusetts Institute of Technology, Cambridge, MA, USA.
8. Ragon Institute of Massachusetts General Hospital, Massachusetts Institute of Technology and Harvard University, Cambridge, MA, USA.
9. Howard Hughes Medical Institute, Chevy Chase, MD, USA.
10. Synthetic Biology Center, Massachusetts Institute of Technology, Cambridge, MA, 02139 USA
11. Department of Electrical Engineering and Computer Science, Massachusetts Institute of Technology, Cambridge, MA, 02139 USA
12. These authors contributed equally: Yonger Xue, Yuebao Zhang, Yichen Zhong.
13. *e-mail: yizhou.dong@mssm.edu

Supplementary Methods

Chemical synthesis of sugar alcohol-derived lipids

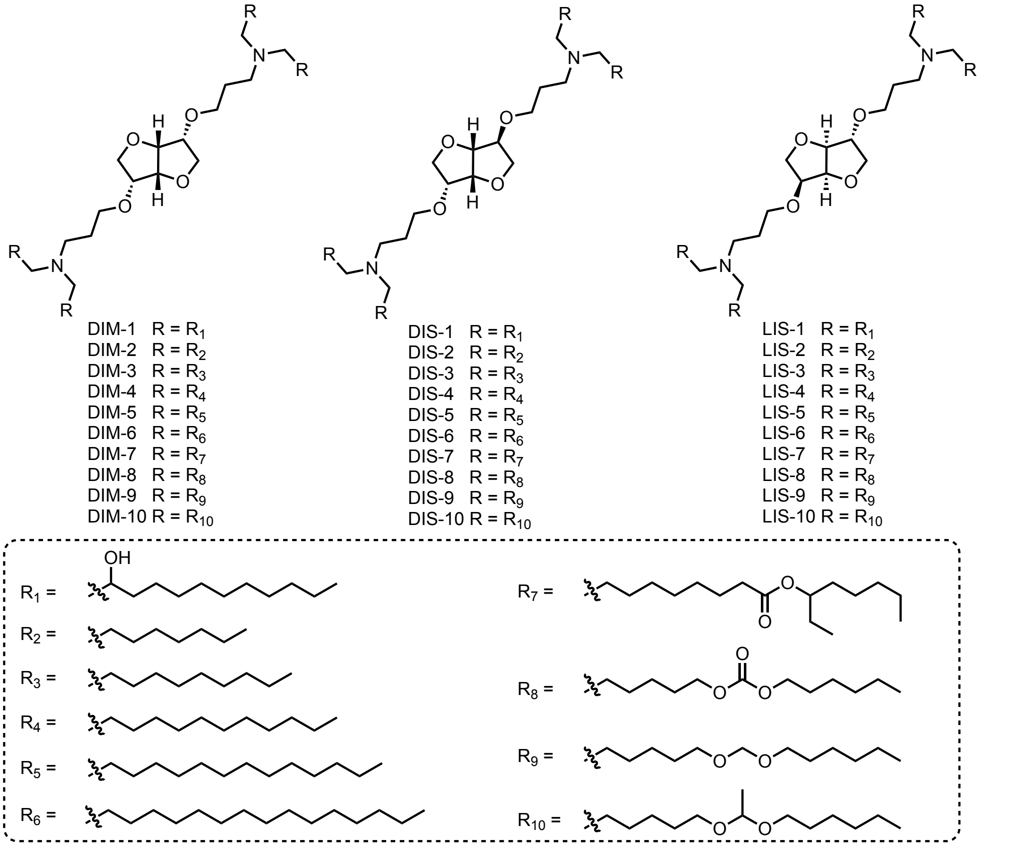

Supplementary Fig.1: Design and structures of sugar alcohol-derived ionizable lipids (DIS, DIM, and LIS series).

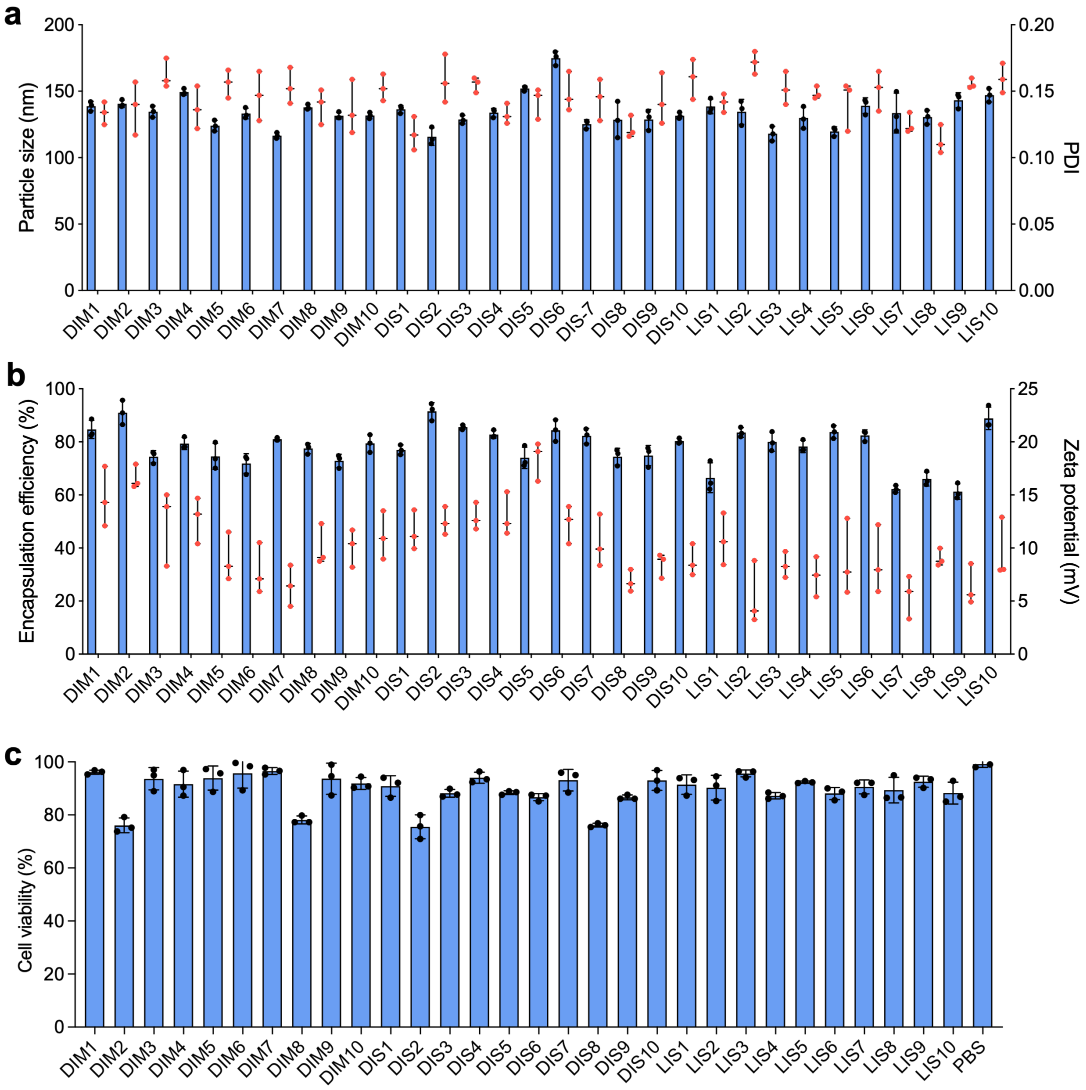

Supplementary Fig. 2: Characterizations of sugar alcohol-derived lipid nanoparticles. **a**, Size and PDI of sugar alcohol-derived LNPs. **b**, Encapsulation efficiency and zeta potential of sugar alcohol-LNPs. **c**, Cytotoxicity of sugar alcohol derived-LNPs in primary ASCs for 24h. Data in **a-c** are from n = 3 biologically independent samples. All data are presented as mean ± s.d.

a

L16 $(4)^4$ orthogonal table

Formulation	Factors (molar ratio)			
	DIM1	DOPE	Chol	DMG-PEG
A	10	20	30	0.5
B	10	30	40	0.75
C	10	40	50	1
D	10	50	60	1.25
E	20	20	40	1
F	20	30	30	1.25
G	20	40	60	0.5
H	20	50	50	0.75
I	30	20	50	1.25
J	30	30	60	1
K	30	40	30	0.75
L	30	50	40	0.5
M	40	20	60	0.75
N	40	30	50	0.5
O	40	40	40	1.25
P	40	50	30	1

b **c**

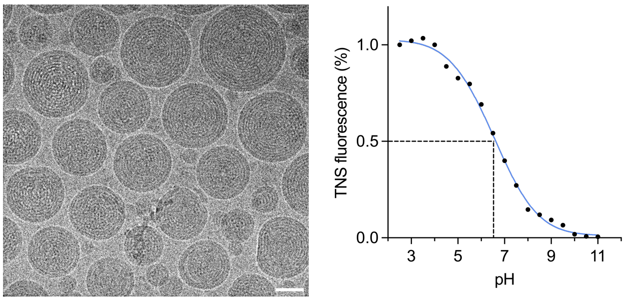

Supplementary Fig. 3: Optimization of DIM1 LNPs. **a**, L16 $(4)^4$ orthogonal table. **b**, Cryo-TEM image of DIM1T encapsulating mRNAs. Scale bar = 50 nm. **c**, Representative TNS assay curves for determining the apparent pK_a of DIM1T LNPs.

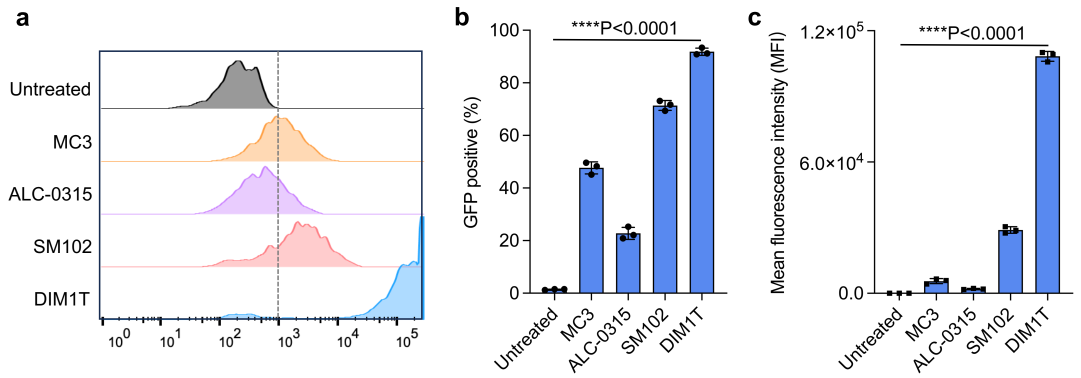

Supplementary Fig. 4: DIM1T LNPs deliver GFP mRNA in ASCs. **a**, GFP expression in ASCs treated with DIM1T LNPs encapsulating GFP mRNA. **b**, Quantification of GFP expression from **a**. **c**, The mean fluorescence intensity (MFI) of GFP in GFP^+ ASCs. Data in **b-c** are from n = 3 biologically independent samples. All data are presented as mean ± s.d. Statistical significance and P values are analyzed by one-way ANOVA followed by Dunnett's multiple comparison test. ****P < 0.0001.

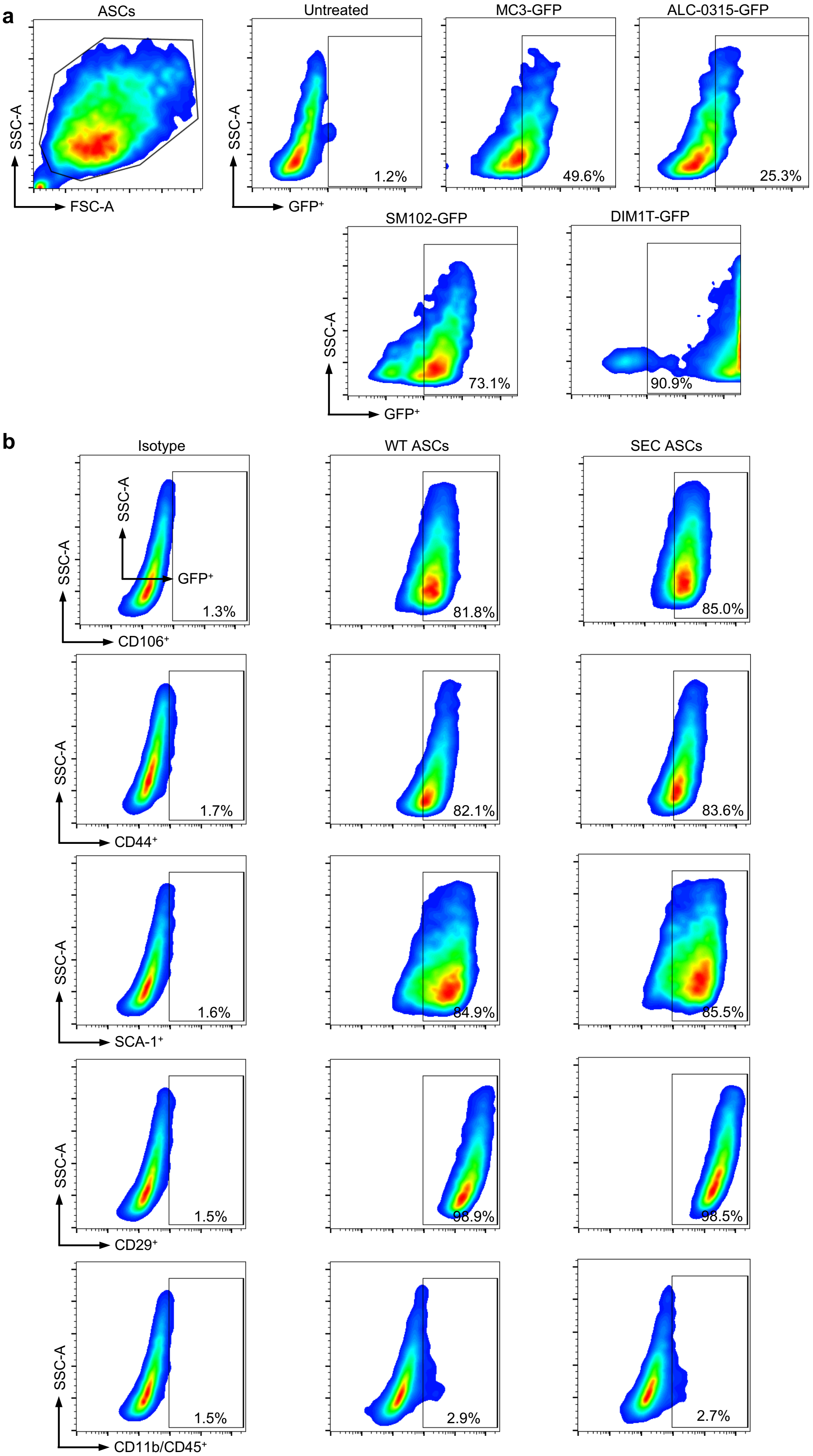

a
ASCs
SSC-A
FSC-A
Untreated
1.2%
GFP+
MC3-GFP
49.6%
ALC-0315-GFP
25.3%
SM102-GFP
73.1%
DIM1T-GFP
90.9%
b
Isotype
WT ASCs
SEC ASCs
1.3%
81.8%
85.0%
CD106+
1.7%
82.1%
83.6%
CD44+
1.6%
84.9%
85.5%
SCA-1+
1.5%
98.9%
98.5%
CD29+
1.5%
2.9%
2.7%
CD11b/CD45+

Supplementary Fig. 5: Flow cytometry gating schemes. **a**, Gating strategies and representative flow cytometry plots to identify GFP^+ ASCs. **b**, Gating strategies and representative flow cytometry plots identify ASC with surface expression of CD106, CD44, SCA-1, CD29 and CD11b/CD45.

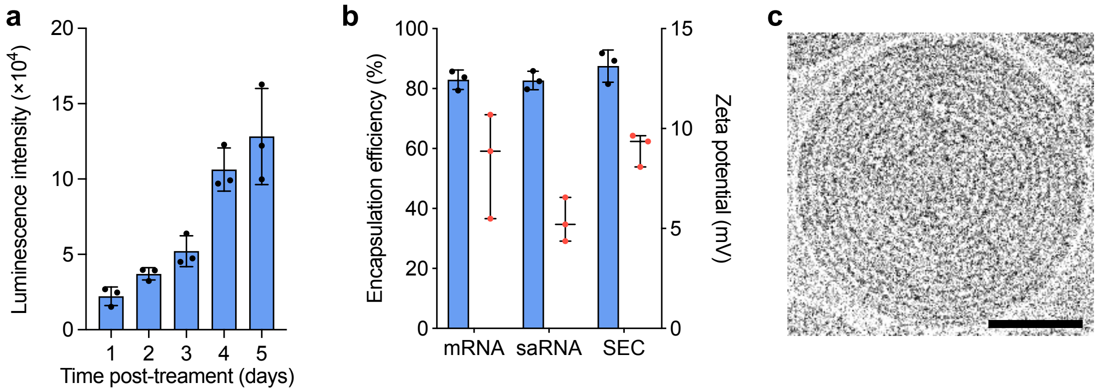

Supplementary Fig. 6: Characterizations of DIM1T LNPs encapsulating SECs. **a**, Luminescence intensity in 293T cells treated with SEC. **b**, Encapsulation efficiency and zeta potential of DIM1T LNPs encapsulating mRNA, saRNA, or SEC. **c**, Cryo-EM image of DIM1T-FLuc SEC LNPs at the saRNA/E3 mRNA mass ratio of 0.5**.** Scale bar = 50 nm. Data in **a-b** are from n = 3 biologically independent samples. All data are presented as mean ± s.d.

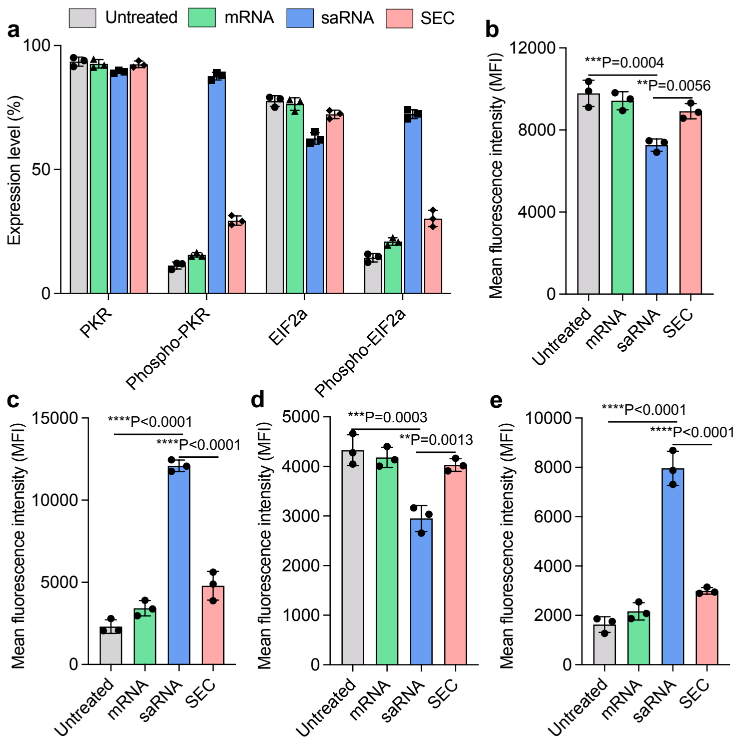

Supplementary Fig. 7: **a**, Expression of the PKR and eIF2α expression in ASCs treated with different formulations at the same total RNA dose 48h post-treatment. The quantification of various biomarkers was analyzed using flow cytometry. **b**, **c**, **d**, **and e**, The mean fluorescence intensity (MFI) of PKR, Phospho-PKR, EIF2α and Phospho-EIF2α in ASCs from **a**. Data in **a-e** are from n = 3 biologically independent samples. All data are presented as mean ± s.d. Statistical significance and P values are analyzed by one-way ANOVA followed by Dunnett's multiple comparison test. **P < 0.01, ***P < 0.001, ****P < 0.0001.

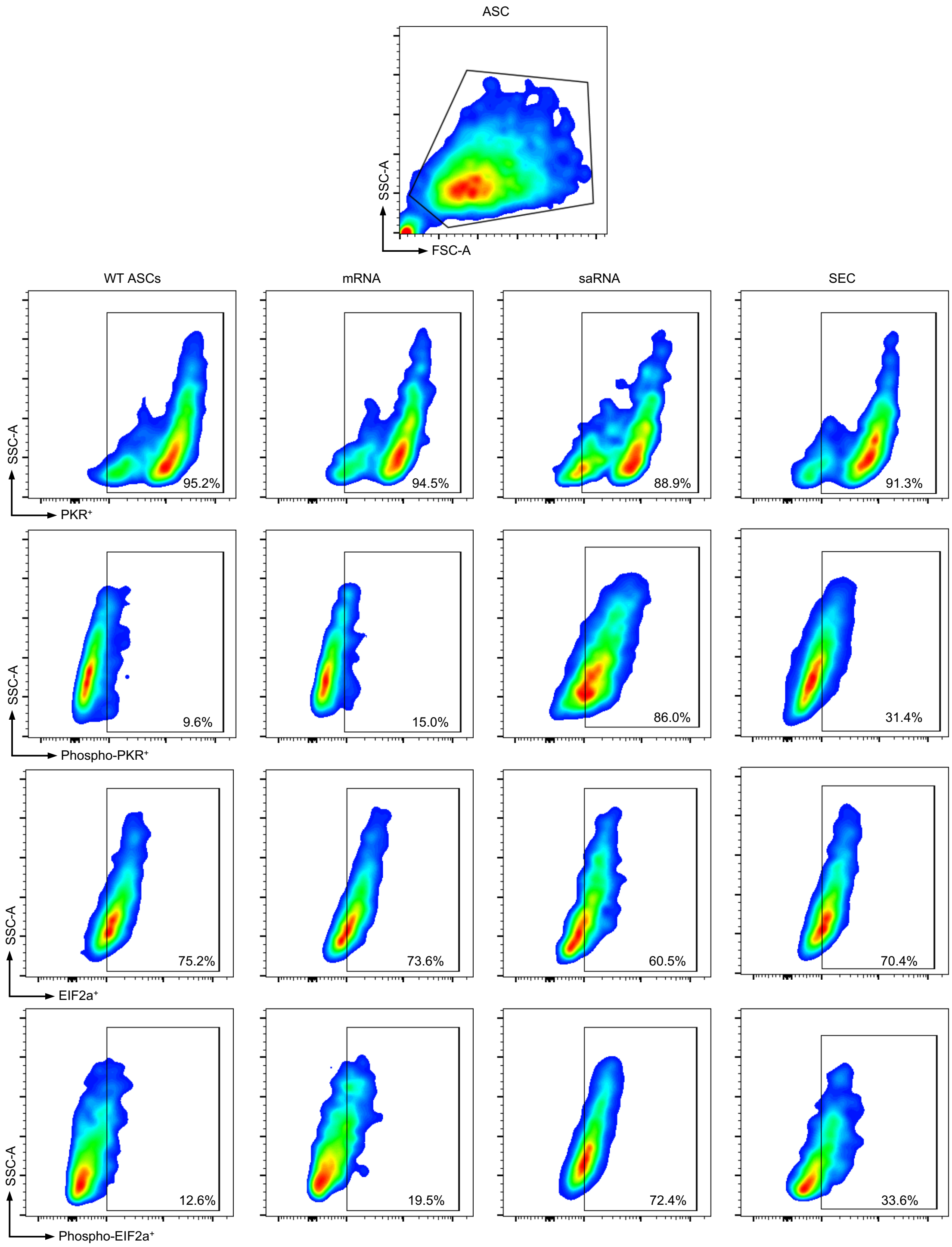

Supplementary Fig. 8: Gating strategies and representative flow cytometry plots identify ASCs of intracellular level of PKR, Phospho-PKR, EIF2a and Phospho-EIF2a.

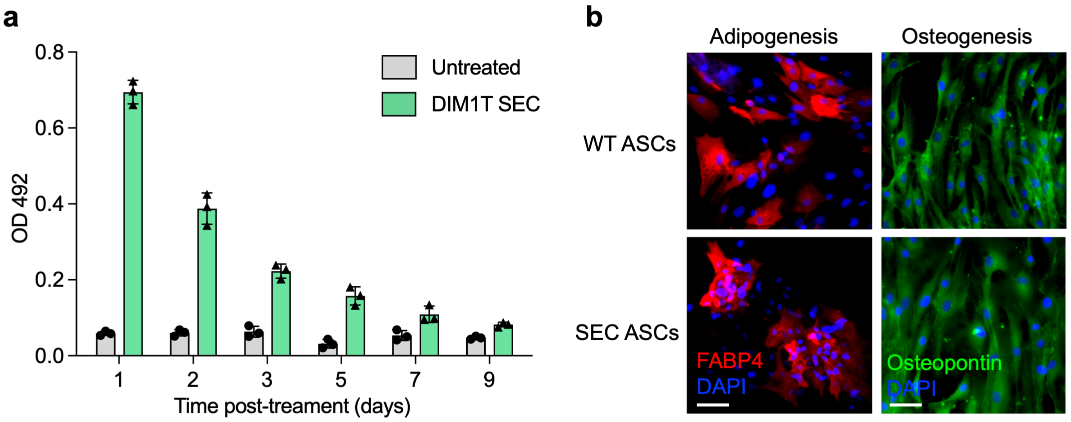

Supplementary Fig. 9: **a**, Expression kinetics of E3 protein delivered by DIM1T LNPs in ASCs. The cell lysate was collected on Days 1, 2, 3, 5, 7, and 9. The E3 protein level in the cell lysates was analyzed using an ELISA assay. **b**, Differentiation capacity of the FLuc DS-ASCs. Blue: DAPI, Red: FABP4^{+}, Green: Osteopontin^{+}. Scale bar = 50 μm. Data in **a** are from n = 3 biologically independent samples. All data are presented as mean ± s.d.

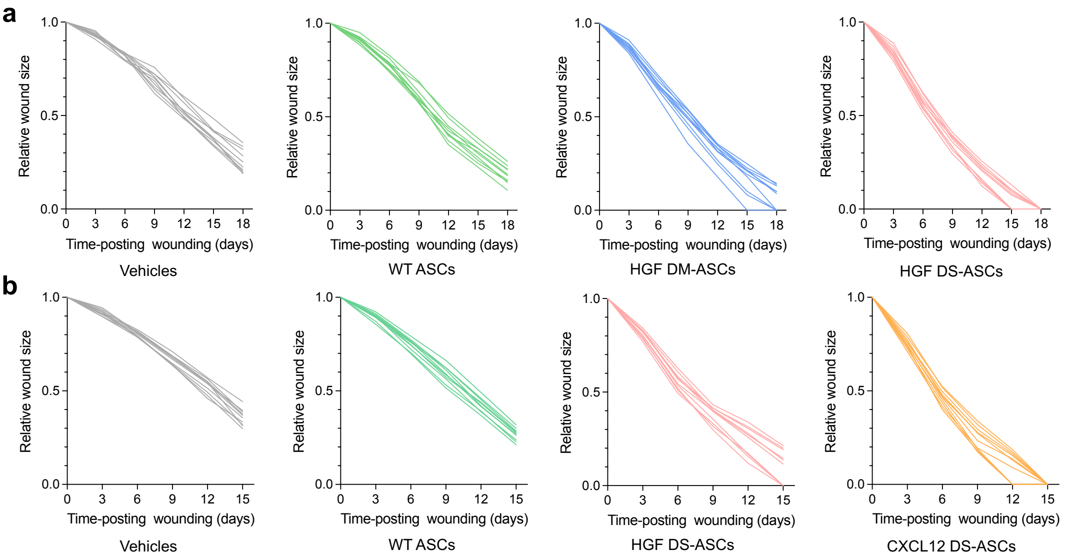

Supplementary Fig. 10: Wound size kinetics of each mouse. **a**, Relative wound size of each mouse from different groups. Gray: Hydrogel vehicle, Green: WT ASCs, Blue: HGF DT-ASCs, Pink: HGF DS-ASCs. **b**, Relative wound size of each wound from different groups. Gray: Hydrogel vehicle, Green: WT ASCs, Pink: HGF DS-ASCs. Yellow: CXCL12 DS-ASCs.

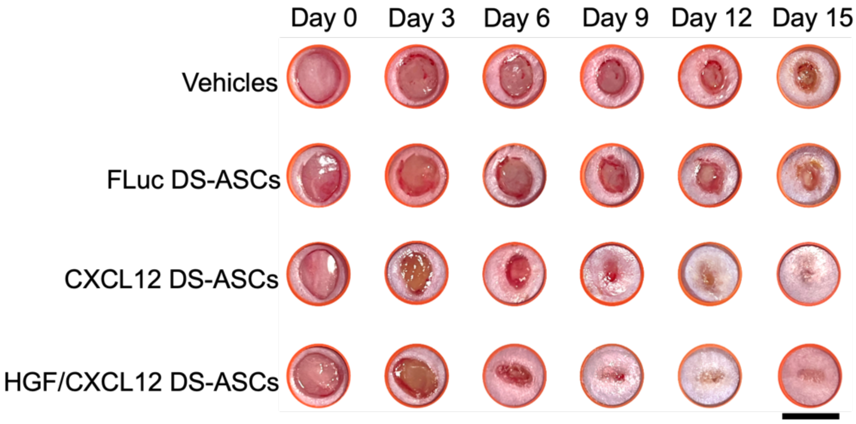

Supplementary Fig. 11: Representative digital images of the wounds of each group from the study on synergistic effects of CXCL12 and HGF in wound healing. Scale bar, 7mm.

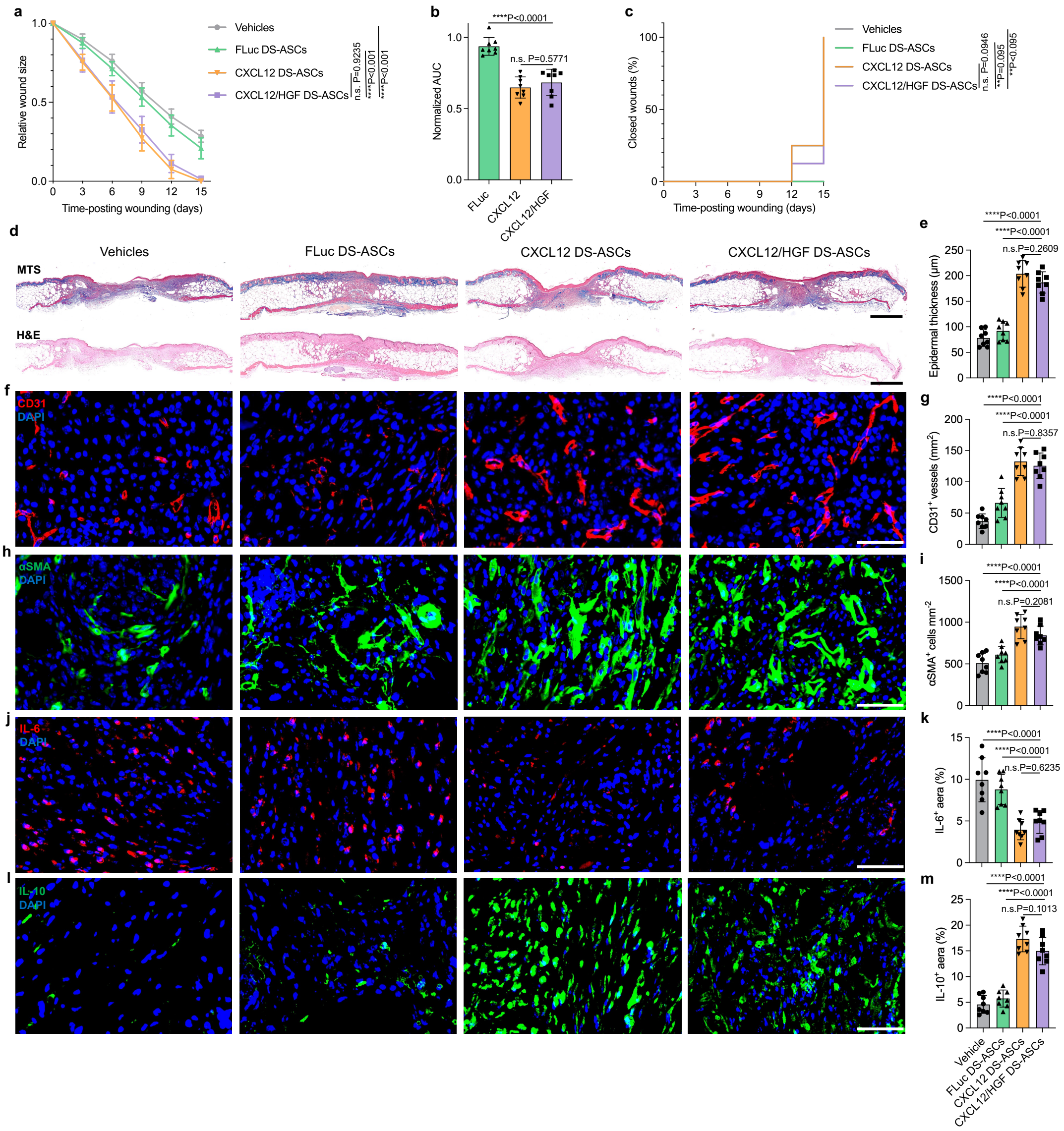

Supplementary Fig. 12: Studies on synergistic effects of CXCL12 and HGF in wound healing. **a**, Relative wound size of mice receiving vehicle controls, FLuc DS-ASCs, CXCL12 DS-ASCs, and CXCL12/HGF DS-ASCs. **b**, Mean AUC of individual wounds of each DS-ASC group normalized to vehicle controls. **c**, The complete wound closure time in vehicle controls, FLuc DS-ASCs, CXCL12 DS-ASCs, and CXCL12/HGF DS-ASCs. The significant differences in time to closure between groups are analyzed using the Log-rank test. **P < 0.01, ****P < 0.0001. **d**, Representative MTS and H&E images of wounds on D15 for each group. **e**, Quantification of the epidermis thickness on the wound tissues from each group. **f**, **h**, **j**, **and l**, Representative CD31$^+$, αSMA$^+$, IL-6 and IL-10 IF images of wounds on D15 for each group. **g**, **i**, **k**, and **m**, Quantification of the CD31$^+$ cells, the αSMA$^+$ cells, the IL-6, and the IL-10. Data in **a-c**, **g**, **i**, **k** and **m** are from n = 8 biologically independent samples. All data are presented as mean ± s.d. Statistical significance and P values are analyzed by one-way ANOVA followed by Dunnett's multiple comparison test. n.s. not significant, P > 0.05, **P < 0.01, ***P < 0.001, ****P < 0.0001. Scale bars, 1 mm (**d**); 50 μm (**f**, **h**, **j**, **and l**).

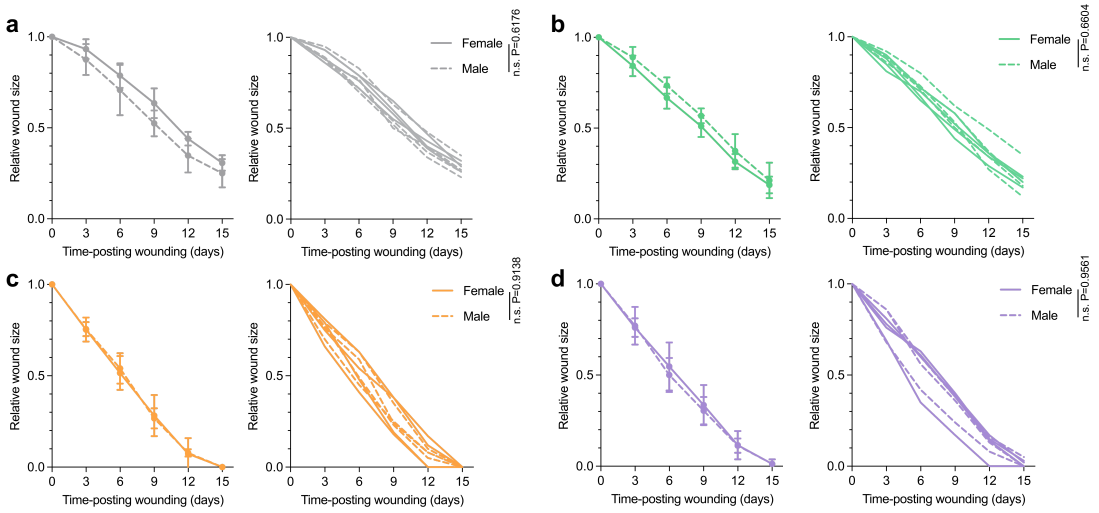

Supplementary Fig. 13: Gender effects on wound healing. **a-d**, Relative wound sizes measured in both male and female mice receiving the following treatments: Vehicle, FLuc DS-ASCs, CXCL12 DS-ASCs, and CXCL12/HGF DS-ASCs, respectively. n = 4 wounds for each gender in every group. All data are presented as mean ± s.d. Statistical significance and P values are analyzed by one-way ANOVA followed by Dunnett's multiple comparison test. n.s. not significant, P> 0.05.

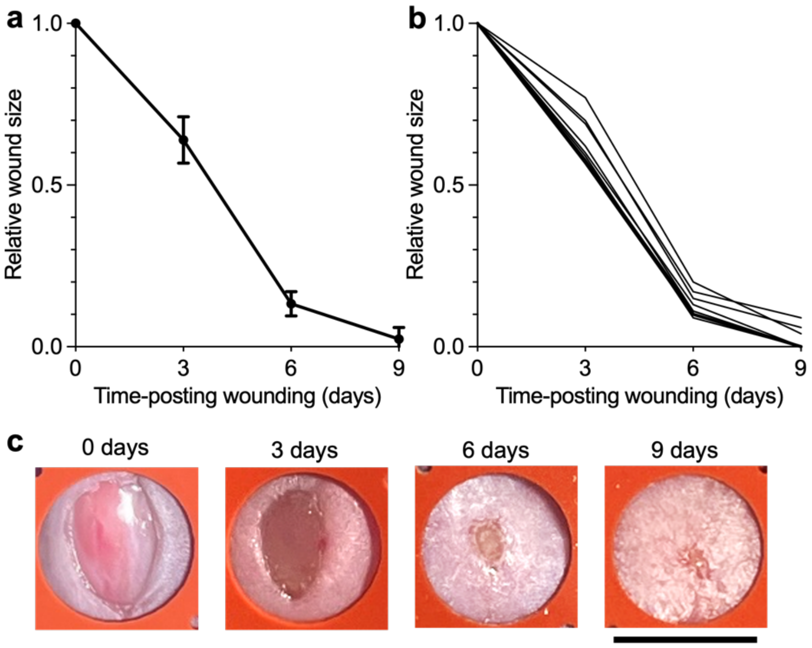

Supplementary Fig. 14: Wound healing kinetics of non-diabetic mice (WT C57BL/6J). **a**, Relative size of the wounds on non-diabetic mice without treatments. n=8 wounds. **b**, Relative size of each wound. **c**, Representative digital images of the wounds. All data are presented as mean ± s.d. Scale bar, 7mm.

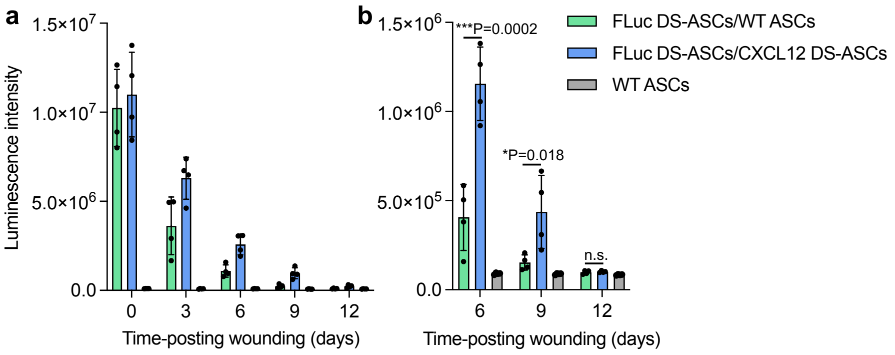

Supplementary Fig. 15: Persistence of DS-ASCs in diabetic wounds. **a**, Luminescence intensity of the embedded FLuc DS-ASC in wounds from Day 0 to Day 12. **b**, Zoom-in plot of luminescence intensity from Day 6 to Day 12 in **a**. Data in **a**-**b** are from n = 4 biologically independent samples. All data are presented as mean ± s.d. Statistical significance and P values are analyzed by one-way ANOVA followed by Dunnett's multiple comparison test. n.s. not significant, P> 0.05, *P < 0.05, ***P < 0.001.

Supplementary Table 1: Coding sequences of mHGF, mCXCL12, and Vaccinia virus (VACV) E3 proteins.

Protein	Coding sequences
mHGF	AUG AUG UGG GGC ACA AAA UUG CUU CCA GUA CUC UUG UUG CA<br>G CAU GUC CUU CUC CAU UUG CUU UUG CUU CAU GUA GCU AUU<br>CCU UAU GCU GAA GGU CAA AAG AAG CGA CGC AAU ACU CUU CA<br>C GAA UUU AAG AAG AGC GCA AAA ACC ACU UUG ACU AAA GAA G<br>AU CCU CUG CUC AAG AUA AAG ACA AAG AAG GUG AAC AGC GCC<br>GAU GAA UGU GCC AAU CGA UGC AUA CGC AAU AGG GGU UUC AC<br>U UUC ACU UGU AAA GCU UUU GUU UUU GAU AAG UCU CGA AAA C<br>GC UGU UAU UGG UAU CCU UUU AAU UCC AUG UCU UCC GGG GU<br>U AAA AAA GGA UUC GGA CAU GAG UUU GAC CUC UAC GAA AAC A<br>AA GAC UAU AUU CGG AAU UGC AUA AUC GGC AAA GGA GGU AGU<br>UAU AAA GGU ACA GUC AGU AUC ACU AAG UCC GGU AUC AAG UG<br>C CAG CCU UGG AAU AGC AUG AUC CCA CAC GAG CAU AGU UUU<br>UUG CCC UCU UCU UAU CGC GGU AAA GAU UUG CAG GAG AAC UA<br>C UGU CGA AAU CCA CGG GGA GAA GAA GGC GGU CCU UGG UGC<br>UUU ACU UCU AAU CCC GAA GUG CGG UAC GAA GUA UGC GAC AU<br>U CCA CAG UGU AGC GAA GUA GAA UGC AUG ACU UGU AAC GGC<br>GAA UCA UAC CGG GGA CCC AUG GAC CAU ACA GAG UCU GGA AA<br>G ACU UGC CAA CGC UGG GAC CAA CAA ACA CCC CAU CGC CAU A<br>AG UUC CUU CCA GAA AGA UAU CCU GAU AAA GGA UUC GAU GAU<br>AAC UAC UGU CGG AAU CCU GAU GGU AAG CCU CGA CCU UGG U<br>GC UAC ACA CUU GAU CCC GAU ACC CCA UGG GAA UAC UGU GCA<br>AUA AAG ACC UGU GCC CAU UCC GCC GUC AAU GAA ACC GAC G<br>UU CCC AUG GAA ACU ACU GAA UGC AUA CAG GGC CAG GGU GAA<br>GGG UAU CGC GGC ACA AGC AAC ACU AUA UGG AAC GGG AUC C<br>CU UGC CAA CGC UGG GAU UCC CAG UAU CCA CAU AAA CAC GAU<br>AUA ACU CCU GAG AAC UUC AAG UGU AAA GAU CUC CGC GAG AA<br>U UAU UGU AGG AAU CCU GAU GGG GCA GAG UCC CCU UGG UGU<br>UUU ACU ACU GAC CCC AAU AUC CGC GUA GGG UAU UGC UCC CA<br>G AUU CCC AAG UGC GAC GUU UCC UCC GGC CAA GAC UGU UAU<br>AGG GGU AAU GGU AAG AAC UAU AUG GGU AAU UUG UCA AAA AC<br>U AGA UCA GGC UUG ACA UGU UCU AUG UGG GAC AAA AAU AUG<br>GAG GAC UUG CAC CGA CAU AUC UUU UGG GAG CCA GAC GCA UC<br>U AAA UUG AAC AAG AAC UAU UGU AGA AAC CCU GAU GAU GAU G<br>CC CAC GGC CCA UGG UGC UAC ACA GGA AAC CCA CUU AUC CCC<br>UGG GAC UAU UGC CCU AUA UCU CGA UGU GAG GGU GAC ACC A<br>CA CCU ACU AUA GUG AAC CUG GAU CAU CCA GUC AUA UCA UGU<br>GCA AAG ACA AAA CAG CUG AGA GUU GUA AAU GGU AUU CCA AC<br>C CAG ACA ACC GUA GGA UGG AUG GUG AGU CUC AAA UAU AGA A<br>AU AAG CAC AUU UGC GGA GGC UCU CUU AUC AAA GAG UCC UGG<br>GUA CUC ACC GCA CGC CAG UGU UUU CCU GCA CGG AAC AAA G<br>AU CUU AAG GAU UAU GAA GCC UGG CUG GGG AUA CAC GAC GUA

	CAU GAA AGG GGA GAA GAG AAG CGA AAA CAA AUU CUU AAC AU<br>C AGC CAG UUG GUG UAC GGG CCU GAA GGA UCC GAU UUG GUG<br>UUG UUG AAA CUG GCU CGG CCC GCC AUC UUG GAC AAU UUC G<br>UC AGC ACU AUA GAC CUU CCC UCA UAC GGU UGU ACU AUU CCC<br>GAA AAA ACC ACC UGU UCA AUC UAU GGC UGG GGA UAC ACA GG<br>G CUG AUC AAU GCU GAC GGU CUC CUG CGA GUU GCA CAU CUU<br>UAC AUC AUG GGU AAC GAG AAG UGU UCC CAA CAU CAC CAA GG<br>U AAA GUA ACU CUC AAC GAA UCA GAA CUC UGC GCU GGG GCU<br>GAG AAA AUC GGG UCC GGC CCU UGU GAA GGA GAC UAC GGC G<br>GA CCA CUU AUC UGC GAG CAG CAC AAA AUG AGA AUG GUU CUG<br>GGU GUC AUA GUU CCC GGG CGC GGU UGC GCC AUU CCU AAC<br>CGA CCA GGU AUU UUU GUC AGG GUG GCC UAU UAC GCC AAG U<br>GG AUU CAC AAG GUA AUU UUG ACU UAC AAA CUU
mCXCL12	AUG GAU GCC AAA GUG GUA GCC GUU UUG GCA CUU GUA UUG G<br>CC GCA CUC UGU AUU UCA GAU GGG AAA CCC GUA AGC CUG AGU<br>UAC AGG UGC CCC UGC CGG UUU UUU GAG UCA CAU AUC GCC C<br>GA GCU AAU GUG AAG CAU UUG AAG AUC CUU AAC ACU CCU AAC<br>UGC GCU CUG CAG AUA GUG GCC AGA CUG AAG AAC AAU AAC CG<br>A CAG GUU UGC AUC GAC CCA AAA CUG AAG UGG AUA CAA GAG U<br>AU CUU GAG AAG GCU CUU AAC AAA AGA CUU AAA AUG
VACV E3	AUG UCU AAA AUC UAU AUC GAU GAG CGA AGC GAU GCC GAA AU<br>A GUU UGU GCA GCC AUA AAG AAC AUU GGC AUU GAA GGG GCC<br>ACU GCC GCU CAG CUC ACA CGC CAG UUG AAC AUG GAG AAA CG<br>C GAA GUA AAC AAG GCA CUU UAU GAC CUG CAG CGA AGU GCA A<br>UG GUU UAC AGC UCU GAC GAU AUA CCU CCC CGC UGG UUU AU<br>G ACA ACC GAA GCU GAU AAG CCC GAU GCU GAU GCU AUG GCA<br>GAC GUU AUC AUC GAU GAU GUU UCA CGC GAG AAG UCA AUG CG<br>A GAA GAC CAC AAG AGU UUC GAU GAC GUG AUU CCC GCA AAG A<br>AA AUC AUC GAC UGG AAG GAU GCC AAC CCU GUG ACC AUA AUA<br>AAC GAA UAU UGC CAA AUC ACA AAA CGG GAC UGG UCU UUU AG<br>A AUA GAA UCU GUU GGG CCU AGC AAU AGU CCA ACC UUU UAC G<br>CU UGU GUA GAC AUC GAC GGC AGG GUU UUC GAC AAG GCA GA<br>C GGC AAA UCA AAA AGA GAU GCU AAG AAC AAU GCU GCU AAA C<br>UU GCU GUG GAU AAG CUG CUC GGU UAU GUC AUC AUU CGG UU<br>U
