# Supplementary material for: LNP-RNA-engineered adipose stem cells for accelerated diabetic wound healing
Source: Nat Commun. 2024 Jan 25;15:739. doi: 10.1038/s41467-024-45094-5 (PMC10811230; doi:10.1038/s41467-024-45094-5)
Supplement: Supplementary file 3 — Reporting Summary [file 41467_2024_45094_MOESM3_ESM.pdf]

nature portfolio

Corresponding author(s): Yizhou Dong

Last updated by author(s): Dec 15, 2023

# Reporting Summary

Nature Portfolio wishes to improve the reproducibility of the work that we publish. This form provides structure for consistency and transparency in reporting. For further information on Nature Portfolio policies, see our Editorial Policies and the Editorial Policy Checklist.

## Statistics

For all statistical analyses, confirm that the following items are present in the figure legend, table legend, main text, or Methods section.

| n/a | Confirmed | |
|---|---|---|
| ☐ | ☒ | The exact sample size (*n*) for each experimental group/condition, given as a discrete number and unit of measurement |
| ☐ | ☒ | A statement on whether measurements were taken from distinct samples or whether the same sample was measured repeatedly |
| ☐ | ☒ | The statistical test(s) used AND whether they are one- or two-sided<br>*Only common tests should be described solely by name; describe more complex techniques in the Methods section.* |
| ☐ | ☒ | A description of all covariates tested |
| ☐ | ☒ | A description of any assumptions or corrections, such as tests of normality and adjustment for multiple comparisons |
| ☐ | ☒ | A full description of the statistical parameters including central tendency (e.g. means) or other basic estimates (e.g. regression coefficient) AND variation (e.g. standard deviation) or associated estimates of uncertainty (e.g. confidence intervals) |
| ☐ | ☒ | For null hypothesis testing, the test statistic (e.g. *F*, *t*, *r*) with confidence intervals, effect sizes, degrees of freedom and *P* value noted<br>*Give P values as exact values whenever suitable.* |
| ☒ | ☐ | For Bayesian analysis, information on the choice of priors and Markov chain Monte Carlo settings |
| ☒ | ☐ | For hierarchical and complex designs, identification of the appropriate level for tests and full reporting of outcomes |
| ☒ | ☐ | Estimates of effect sizes (e.g. Cohen's *d*, Pearson's *r*), indicating how they were calculated |

*Our web collection on statistics for biologists contains articles on many of the points above.*

## Software and code

Policy information about availability of computer code

| | |
|---|---|
| Data collection | Living Image®, Thermo Scientific EPU software, Nikon A1R Live Cell Imaging Confocal Microscope, LSRFortessa Flow Cytometer (BD Biosciences), BioTek CYTATION 5 plate reader, Malvern Zetasizer NanoZS, and Microsoft Excel (Version 2112) |
| Data analysis | GraphPad Prism 9, Microsoft Excel (Version 2112), and FlowJo (Version 10.4). |

For manuscripts utilizing custom algorithms or software that are central to the research but not yet described in published literature, software must be made available to editors and reviewers. We strongly encourage code deposition in a community repository (e.g. GitHub). See the Nature Portfolio guidelines for submitting code & software for further information.

## Data

Policy information about availability of data

All manuscripts must include a data availability statement. This statement should provide the following information, where applicable:

- Accession codes, unique identifiers, or web links for publicly available datasets
- A description of any restrictions on data availability
- For clinical datasets or third party data, please ensure that the statement adheres to our policy

All experimental data have been included in the manuscript and supplementary information. Source data are provided with this paper.

## Research involving human participants, their data, or biological material

Policy information about studies with human participants or human data. See also policy information about sex, gender (identity/presentation), and sexual orientation and race, ethnicity and racism.

| | |
|---|---|
| Reporting on sex and gender | N/A |
| Reporting on race, ethnicity, or other socially relevant groupings | N/A |
| Population characteristics | N/A |
| Recruitment | N/A |
| Ethics oversight | N/A |

Note that full information on the approval of the study protocol must also be provided in the manuscript.

# Field-specific reporting

Please select the one below that is the best fit for your research. If you are not sure, read the appropriate sections before making your selection.

[x] Life sciences [ ] Behavioural & social sciences [ ] Ecological, evolutionary & environmental sciences

For a reference copy of the document with all sections, see nature.com/documents/nr-reporting-summary-flat.pdf

# Life sciences study design

All studies must disclose on these points even when the disclosure is negative.

| | |
|---|---|
| Sample size | A minimum of 3 biological repeats is applied to evaluate significance and to calculate statistical descriptions. The sample size 4-5 mice per group, 2 wounds per mouse) for in vivo studies was used on the basis of published literature on similar evaluations (Nat Commun. 2020; 11: 2470.) and was selected to minimize the number of animals and meanwhile obtain statistical validity based on power analysis. |
| Data exclusions | No data were excluded. |
| Replication | In vitro experiments were repeated independently for at least 3 times. In vivo experiments were repeated independently for at least 2 times and on different models (n = 8 or 10 biological replicates for each group). |
| Randomization | Samples were randomly allocated to corresponding experimental groups. |
| Blinding | Researchers were not blinded for lipid synthesis, mRNA production, nanoparticle formulation and cell assay because the investigators had to take careful of different experimental conditions. However, these experiments including material synthesis, particle formulation, flow assays and animal experiments were performed by multiple researchers, who had minimal information of sample identification. |

# Reporting for specific materials, systems and methods

We require information from authors about some types of materials, experimental systems and methods used in many studies. Here, indicate whether each material, system or method listed is relevant to your study. If you are not sure if a list item applies to your research, read the appropriate section before selecting a response.

### Materials & experimental systems

| n/a | Involved in the study | |
|---|---|---|
| [ ] | [x] | Antibodies |
| [ ] | [x] | Eukaryotic cell lines |
| [x] | [ ] | Palaeontology and archaeology |
| [ ] | [x] | Animals and other organisms |
| [x] | [ ] | Clinical data |
| [x] | [ ] | Dual use research of concern |
| [x] | [ ] | Plants |

### Methods

| n/a | Involved in the study | |
|---|---|---|
| [x] | [ ] | ChIP-seq |
| [ ] | [x] | Flow cytometry |
| [x] | [ ] | MRI-based neuroimaging |

## Antibodies

| | |
|---|---|
| Antibodies used | Antibodies used for flow cytometry:<br>FITC anti-mouse CD106 Antibody (BioLegend, 105705), 1: 100 dilution; |

| | |
|---|---|
| | FITC anti-mouse/human CD44 Antibody (BioLegend, 103005), 1: 100 dilution;<br>FITC anti-mouse Ly-6A/E (Sca-1) Antibody (BioLegend, 108105), 1: 100 dilution;<br>FITC anti-mouse/rat CD29 Antibody (BioLegend, 102205), 1: 100 dilution;<br>FITC anti-mouse/human CD11b Antibody (BioLegend, 101205), 1: 100 dilution;<br>FITC anti-mouse CD45 Antibody (BioLegend, 157213), 1: 100 dilution;<br>Recombinant Anti-PKR antibody [EPR19374] (Abcam, ab184257), 1: 100 dilution;<br>PKR (Thr446 + Thr451) Antibody (Bioss, BS-3337R), 1: 100 dilution;<br>EIF2S1/EIF2A Polyclonal antibody (Proteintech, CL488-11170), 1: 50 dilution;<br>Phospho-EIF2S1 (Ser51) Monoclonal antibody (Proteintech, CL488-68023).1: 50 dilution;<br><br>Antibodies used for ELISA:<br>Rabbit anti-Vaccinia virus (strain Copenhagen) (VACV) E3L Polyclonal antibody (CUSABIO, CSB-PA322729ZA01VAA), 1: 2000 dilution<br><br>Antibodies used for IF:<br>Anti-mouse IL-6 Ab (Abcam, ab290735), 1:50 dilution;<br>Anti-mouse IL-10 Ab (Abcam, ab9969), 1:200 dilution;<br>Anti-mouse CD31 Ab (Abcam, ab222783), 1:200 dilution;<br>Anti-mouse aSMA Ab (Abcam, ab7817), 1:200 dilution;<br><br>Secondary Antibodies:<br>Alexa Fluor 594 donkey anti-rabbit IgG (Invitrogen, A21207), 1:200;<br>Alexa Fluor 488 donkey anti-rabbit IgG (Invitrogen, A21206), 1:800; |
| Validation | These antibodies have been verified by the supplier and reported for the use in flow cytometry, ELISA and IF. All the antibodies used are from commercial sources and validation data are available on the manufacturer's website based on their catalogue numbers. The manufacturers include, Invitrogen, Bioss, Biolegend, Cusabio, Abcam and Proteintech. |

# Eukaryotic cell lines

Policy information about cell lines and Sex and Gender in Research

| | |
|---|---|
| Cell line source(s) | Mouse adipose-derived stem cells were obtained by adaptation of previous procedures. 293T cell line was purchased from American Type Culture Collection (ATCC). |
| Authentication | Cell lines were not independently authenticated. |
| Mycoplasma contamination | Cell lines do not have mycoplasma contamination. |
| Commonly misidentified lines<br>(See ICLAC register) | No commonly misidentified cell lines were used. |

# Animals and other research organisms

Policy information about studies involving animals; ARRIVE guidelines recommended for reporting animal research, and Sex and Gender in Research

| | |
|---|---|
| Laboratory animals | All female and male db/db mice (BKS.Cg-Dock7m +/+ Leprdb/J, 00642, 10-12 weeks) as well as male C57BL/6 mice (6-8 weeks) were purchased from the Jackson Laboratory. All mice were housed in The Ohio State University (2014A00000106) or in The Icahn School of Medicine at Mount Sinai (IPROTO202200000134). All mouse studies were approved by the Institutional Animal Care and Use Committee (IACUC) and complied with local, state, and federal regulations. Mice were housed under a barrier environment (~20°C, ~45% humidity, and 12/12 light/dark cycle). After wound generation, up to 2 mice were housed in each cage. |
| Wild animals | The study did not involve wild animals. |
| Reporting on sex | Both female and male db/db mice were used in the animal studies. The analysis of relative wound size data indicated that gender differences did not significantly impact the rates of wound healing in diabetic mice. |
| Field-collected samples | The study did not involve samples collected from field. |
| Ethics oversight | All mouse studies were approved by the Institutional Animal Care and Use Committee at The Ohio State University and The Icahn School of Medicine at Mount Sinai. |

Note that full information on the approval of the study protocol must also be provided in the manuscript.

## Plants

| | |
|---|---|
| Seed stocks | N/A |
| Novel plant genotypes | N/A |
| Authentication | N/A |

## Flow Cytometry

### Plots

Confirm that:

- [x] The axis labels state the marker and fluorochrome used (e.g. CD4-FITC).
- [x] The axis scales are clearly visible. Include numbers along axes only for bottom left plot of group (a 'group' is an analysis of identical markers).
- [x] All plots are contour plots with outliers or pseudocolor plots.
- [x] A numerical value for number of cells or percentage (with statistics) is provided.

### Methodology

| | |
|---|---|
| Sample preparation | The adipose stem cells were stained by antibodies based on their corresponding protocols. For intracellular staining, the cells were treated by fixation and permeation buffers before antibody staining. |
| Instrument | LSRFortessa Flow Cytometer (BD Biosciences) |
| Software | FlowJo (Version 10.4) |
| Cell population abundance | The cell abundance was shown in the results. |
| Gating strategy | Cells was gated on FSC/SSC scatter. |

- [ ] Tick this box to confirm that a figure exemplifying the gating strategy is provided in the Supplementary Information.
